# Real-world effectiveness of peginterferon α-2b plus ribavirin in a Canadian cohort of treatment-naïve chronic hepatitis C patients with genotypes 2 or 3: results of the PoWer and RediPEN studies

**DOI:** 10.1007/s10096-016-2576-1

**Published:** 2016-02-06

**Authors:** P. Marotta, R. Bailey, M. Elkashab, J. Farley, S. V. Feinman, K. Peltekian, M. Poliquin, H. Witt-Sullivan, E. Rampakakis, M. Drolet, C. Cooper

**Affiliations:** London Health Sciences Center, London, ON Canada; University of Alberta, Edmonton, AB Canada; Toronto Liver Centre, Toronto, ON Canada; Dr. John Farley Inc., Vancouver, BC Canada; Mount Sinai Hospital, Toronto, ON Canada; Atlantic Hepatology Services, Halifax, NS Canada; Clinique Médicale L’Actuel, Montreal, QC Canada; McMaster University, Hamilton, ON Canada; JSS Medical Research Inc., St-Laurent, QC Canada; Merck Canada Inc., Kirkland, QC Canada; Ottawa Hospital Research Institute, Ottawa, ON Canada

## Abstract

The purpose of this investigation was to assess the real-life effectiveness of pegylated interferon (peg-IFN) α-2b with ribavirin (RBV) in a cohort of treatment-naïve patients with chronic genotypes 2 (G2) or 3 (G3) hepatitis C virus (HCV) infection. A post-hoc pooled analysis of two Canadian multicenter, observational studies, RediPEN and PoWer, was carried out. A total of 1242 G2- or G3-infected patients were included. The primary outcome was sustained virologic response (SVR). Secondary endpoints included early virologic response (EVR), end-of-treatment (EOT) response, and relapse. Multivariate logistic regression was used to identify independent predictors of treatment response. SVR in G2 and G3 was 74.4 % and 63.6 %, respectively. Relapse occurred in 12.7 % and 19.1 % of G2- and G3-infected patients achieving EOT response, respectively. Overall, G3 was found to independently predict reduced SVR [odds ratio (OR) = 0.20; *p* = 0.007] and increased relapse (OR = 6.84; *p* = 0.022). Among G3-infected patients, increasing fibrosis score was the most important factor predicting reduced SVR [F2 vs. F0/F1 (OR = 0.41; *p* = 0.009); F3 vs. F0/F1 (OR = 0.72; *p* = 0.338); F4 vs. F0/F1 (OR = 0.27; *p* = 0.001)]. Male gender (OR = 13.16; *p* = 0.020) and higher fibrosis score [F2 vs. F0/F1 (OR = 9.72; *p* = 0.016); F3/F4 vs. F0/F1 (OR = 4.23; *p* = 0.113)] were associated with increased relapse in G3 patients. These results support the real-life effectiveness of peg-IFN α-2b plus ribavirin in HCV G2- and G3-infected patients. Overall, genotype was identified as the most significant predictor of treatment outcome. Fibrosis score and gender were key outcome predictors in the G3-infected population. In clinical settings, peg-INF/RBV offers an alternative for patients without access to all oral direct-acting antivirals.

## Introduction

According to the World Health Organization (WHO), 2–3 % of the world’s population is currently infected with the hepatitis C virus (HCV) [1]. The epidemiology of HCV is highly variable, with prevalence in individual countries ranging from <1 % to >10 % [2]. In 2013, the prevalence of HCV in Canada was estimated at 251,990 viremic individuals, with the highest number of cases occurring in the 40–54 years age group [3]. Genotypes 1 (G1; 64.1 %), 2 (G2; 14.1 %), and 3 (G3; 20.2 %) account for over 98 % of cases. Overall, approximately 8000 new cases are diagnosed each year, of which an estimated 70–80 % will progress to chronic infection [4, 5]. Moreover, the reported cases are likely an underestimate of the true incidence of HCV infection, as a majority of acute HCV infections are asymptomatic and, therefore, go undetected [6].

Due to similar rates of viral decline and significantly higher rates of sustained virologic response (SVR) compared with G1 infections, G2 and G3 have traditionally been considered a homogeneous entity, grouped together in efficacy analyses [7]. However, evidence has emerged supporting the differentiation of G2 and G3 in terms of clinical presentation and response to treatment. Several reports have indicated that patients infected with G3 experience higher rates of steatosis and bile duct lesions than their non-G3-infected counterparts [8–10], with steatosis more significantly reduced by treatment among patients infected with G3 than in other genotypes [8]. Furthermore, although still higher than in G1-infected patients, pegylated interferon-α (peg-IFN α) + ribavirin dual combination therapy (PEG/RBV) in patients infected with G3 has been associated with lower rates of SVR and higher rates of relapse compared to those observed in patients infected with G2 [7, 11–17].

Up until recently, European and North American treatment guidelines have dictated first-line PEG/RBV combination therapy in both G2- and G3-infected patients [18–20]. However, based on the results of recent phase III trials [21–23], Canadian and US recommendations have shifted focus to sofosbuvir (SOF), an HCV polymerase inhibitor, in combination therapy with ribavirin as the first-line treatment of G2 and G3 [24, 25]. Despite the promise of SOF-containing treatment regimens, the cost of SOF is a significant barrier preventing universal access. Moreover, the results of several recent cost effectiveness analyses have shown that, despite high rates of SVR, lower treatment duration, and a lower incidence of adverse events, the incremental cost-effectiveness ratio (ICER) of SOF-based treatments at their current cost, when compared to PEG/RBV, exceed willingness-to-pay (WTP) thresholds of up to $100,000 USD [26–29].

By evaluating, in a large cohort of treatment-naïve, chronic G2 or G3 HCV-infected patients followed in Canadian routine clinical care, we sought to clarify the real-life effectiveness of PEG/RBV for the treatment of G2 and G3. Furthermore, we identified genotype-specific predictors of response to treatment.

## Methods

This was a post-hoc analysis of two Canadian open-label, multicenter, observational studies, the PEGETRON® Prospective Optimal Weight-Based Dosing (OW-BD) Response Program (PoWer) and the PEGETRON® REDIPEN® Prospective OW-BD Response Program (RediPEN).

### PoWer

The PoWer program was designed to prospectively assess the real-world SVR rate (defined as HCV RNA undetectable at 24 weeks from the end of treatment) in Canadian HCV patients of all genotypes receiving weight-based ribavirin plus peg-IFN α-2b (PEGETRON® OW-BD). Enrolment took place between December 2002 and August 2005, to a total of 2250 patients. Eligible patients included treatment-naïve adults (>18 years of age), with chronic HCV infection, who were eligible as per the PEGETRON® product monograph for treatment with weight-based ribavirin plus peg-IFN α-2b, and were able to obtain reimbursement for their treatment. Patients were excluded if they had any of the PEGETRON product monograph dictated contraindications pertaining to: hypersensitivity to any interferons, ribavirin, or any component of the injection and/or capsule; pregnant women or males with pregnant female partners; patients with autoimmune hepatitis or a history of autoimmune disease; patients with a history of or a pre-existing severe psychiatric condition; patients with a pre-existing thyroid abnormality; patients with severe renal dysfunction; patients with decompensated liver disease; epileptic patients; patients with a history of severe or unstable pre-existing cardiac disease; patients with hemoglobinopathies. Treatment was as per the PEGETRON® product monograph and the standard of care at each individual participating site. PEGETRON® (peg-IFN α-2b) powder for solution was administered subcutaneously at a dosage of 1.5 mcg/kg/week using plastic disposable syringes, whereas PEGETRON® (ribavirin) capsules (200 mg/capsule) were administered orally twice daily with morning and evening meals. The dose of PEGETRON® (ribavirin) capsules to be used in combination with PEGETRON® (peg-IFN α-2b) powder for solution was based on the patient body weight: patients <64 kg were prescribed a daily dose of 800 mg of ribavirin (four capsules total; two morning, two evening), patients 64 to <85 kg were prescribed a daily dose of 1000 mg of ribavirin (five capsules daily; two morning, three evening), and patients >85 kg were prescribed a daily dose of 1200 mg of ribavirin (six capsules daily; three morning, three evening). The recommended treatment duration was one year, with duration tailored to baseline disease characteristics, response to therapy, and tolerance of the regimen. However, a stopping rule was implemented for patients for whom a virologic response (undetectable HCV RNA) had not occurred by 6 months of treatment, as it was deemed unlikely to occur after this time. Recommended patient assessments were at baseline, end-of-treatment (EOT), as well as 24 weeks following the last dose of the study drug. The primary effectiveness outcome measure of the study was the SVR rate at 24 weeks post-therapy. In addition, EOT response (defined as undetectable HCV RNA at EOT) and relapse (defined as detectable HCV RNA levels within 24 weeks after achieving EOT response) were also evaluated.

### RediPEN

The RediPEN study assessed the real-world effectiveness of weight-based ribavirin plus peg-IFN α-2b (PEGETRON® OW-BD), administered with the REDIPEN® delivery system, in Canadian patients of all genotypes with chronic HCV infection. Additional objectives included the assessment of the dropout rate and the rate of treatment discontinuation due to an adverse event. Eligibility criteria were consistent with those in PoWer, with the exception that patients were treated with PEGETRON® REDIPEN™ and that HBsAg-positive patients, human immunodeficiency virus (HIV)-positive patients, and liver transplant recipients were not eligible for enrolment. In all, 1302 patients were enrolled in RediPEN from July 2005 to June 2008. Treatment was per the PEGETRON® product monograph and the standard of care at each individual participating site. PEGETRON® (peg-IFN α-2b) powder for solution was administered subcutaneously at a dosage of 1.5 mcg/kg/week using the REDIPEN® Single-dose Delivery System, and equivalent weight-based algorithms for ribavirin dosing, as previously described for PoWer, were applied. The treatment duration was 24 or 48 weeks, and was to be individualized as per the judgment of the treating physician based on the baseline disease characteristics, response to therapy, and tolerance of the regimen. However, as in PoWer, a 24-week stopping rule was implemented if virologic response had not been achieved. Recommended patient assessments were at baseline, week 12, EOT (24 or 48 weeks), and 24 weeks following the last dose of the study drug. Effectiveness outcome measures included: early virologic response (EVR; defined as either a >2 log decrease in HCV RNA from baseline or as undetectable HCV RNA between weeks 10 and 14), EOT response, SVR, and relapse.

All patients provided informed consent before any study-related procedure, and both PoWer and RediPEN were conducted as per Good Clinical Practices and the tenets of the Declaration of Helsinki. Ethics approval was obtained by a central Institutional Review Board and local committees, as required for each participating site.

### Pooled analysis

For the purpose of the current post-hoc analysis, concerned with treatment response to PEG/RBV in G2 and G3 HCV-infected patients, the pooled results of both studies in the overall (ITT) and per genotype (G2 and G3) populations are reported. In accordance with the exclusion of HBsAg-positive, HIV-positive, and liver transplant patients from RediPEN, patients in PoWer with these conditions were excluded from the current analysis. As a result, the overall ITT population consisted of 1242 patients, including 468 G2-infected (*n* = 298 originating from PoWer, *n* = 170 from RediPEN) and 774 G3-infected (*n* = 431 originating from PoWer, *n* = 343 from RediPEN) individuals. The primary effectiveness measure was SVR, while secondary outcomes were EVR (available for RediPEN patients only), EOT response, and relapse. Adverse events leading to treatment discontinuation were also assessed (RediPEN patients only). Given that virologic response data at week 4 were not collected in either study, analysis of rapid virologic response (RVR) was not possible. The following parameters were examined as potential predictors of SVR and relapse: genotype, gender, age, race, weight, Metavir fibrosis score, baseline viral load, and geographic region. In addition, the association of fibrosis score and EVR and EOT response was also assessed. Patients lost to follow-up or with missing EVR, EOT response, and SVR information were considered as non-responders.

In the current analysis, descriptive statistics were produced for all variables. For continuous variables, these included the mean, median, minimum, maximum, as well as the standard deviation (SD), while frequency distributions were used for categorical variables. Between-group differences in continuous variables were assessed for statistical significance with the Wilcoxon rank-sum test, while the Pearson chi-squared or Fisher’s exact test, as required, were used for categorical variables. Determinants of response to treatment were assessed using univariate logistic regression. Variables that showed a statistical trend (*p* < 0.150) in the univariate analysis were considered in multivariate analysis to identify independent predictors of response. All analyses were conducted using SAS 9.2 (SAS Institute, Cary, NC).

## Results

### Study patients

Among the 1242 patients included in the ITT population, 468 (37.7 %) were infected with G2 and 774 (62.3 %) were infected with G3. Table 1 summarizes the number of patients included in each analysis, as well as the number discontinuing treatment prematurely. A total of 190 (15.3 %) individuals were discontinued, with comparable rates between genotypes (G2: 14.3 %; G3: 15.8 %). The reason for discontinuation was missing for the majority of patients (collected in RediPEN only); the predominant reasons for discontinuation included adverse events (ITT: *n* = 34; G2: *n* = 15; G3: *n* = 19), loss to follow-up (ITT: *n* = 23; G2: *n* = 5; G3: *n* = 18), and patient choice (ITT: *n* = 11; G2: *n* = 3; G3: *n* = 8).

**Table 1:** Patient disposition

	ITT population (*n* = 1242)	Genotype 2 (*n* = 468)	Genotype 3 (*n* = 774)
	*n* (%)	*n* (%)	*n* (%)
Included in EVR analysis^a^	513 (41.3)	170 (36.3)	343 (44.3)
Included in EOT analysis	1242 (100.0)	468 (100.0)	774 (100.0)
Included in SVR analysis^b^	1242 (100.0)	468 (100.0)	774 (100.0)
Included in relapse analysis	982 (79.0)	386 (82.4)	596 (77.0)
Discontinued	190 (15.3)	67 (14.3)	123 (15.8)
Reasons for discontinuation^a,c^			
Adverse event	34 (17.9)	15 (22.4)	19 (15.4)
Lack of virologic response	2 (1.1)	0 (0.0)	2 (1.6)
Assigned shorter therapy	1 (0.5)	0 (0.0)	1 (0.8)
Lost to follow-up	23 (12.1)	5 (7.5)	18 (14.6)
Patient choice	11 (5.8)	3 (4.5)	8 (6.5)
Geographic reasons	3 (1.6)	1 (1.5)	2 (1.6)
Substance abuse	5 (2.6)	0 (0.0)	5 (4.1)
Non-compliance	4 (2.1)	0 (0.0)	4 (3.3)
Consent withdrawal	1 (0.5)	0 (0.0)	1 (0.8)
Other	1 (0.5)	1 (1.5)	0 (0.0)
Not available	105 (55.2)	42 (62.6)	63 (51.2)

*ITT* intention-to-treat; *EVR* early virologic response; *EOT* end-of-treatment; *SVR* sustained virologic response

^a^Data available for RediPEN only; *n* = 513

^b^Due to variable follow-up, if information at 24 weeks post-EOT was unavailable for the evaluation of SVR24, follow-up information ≥12 weeks post-EOT was utilized for SVR assessment

^c^Proportion of discontinuation based on the total number of patients discontinued per population subgroup

The patient characteristics are provided in Table 2. Information regarding age, gender, and race was available for RediPEN patients only. The mean (SD) age of the ITT population was 45.4 years (10.1), where the majority of patients (58.5 %) were between the ages of 40 and 54 years. The majority of patients were male (62.0 %), Caucasian (82.3 %), and weighed in excess of 75 kg (59.7 %). In descending order, patients were enrolled primarily in Ontario (46.8 %), followed by British Columbia (20.6 %), the Prairie provinces (15.1 %), Quebec (14.7 %), and Atlantic Canada (2.7 %). No remarkable differences were observed between genotypes, with the exception of age, which was higher in G2-infected patients (49.6 vs. 43.4 years).

**Table 2:** Patient characteristics

Characteristic	ITT population (*n* = 1242)	Genotype 2 (*n* = 468)	Genotype 3 (*n* = 774)
Genotype (G), *n* (%)^a^			
2	468 (37.7)	468 (100)	
3	774 (62.3)		774 (100)
Total	1242 (100.0)		
Gender, male, *n* (%)^b^	318 (62.0)	101 (59.4)	217 (63.3)
Age, years^b^			
Mean (SD)	45.4 (10.1)	49.6 (9.4)	43.4 (9.9)
Median	45.5	50.8	43.8
Range	18.4–76.8	21.1–76.8	18.4–70.6
Age categories, *n* (%)^b^			
<25	16 (3.1)	3 (1.8)	13 (3.8)
25–39	122 (23.8)	19 (11.2)	103 (30.0)
40–54	300 (58.5)	109 (64.1)	191 (55.7)
≥55	75 (14.6)	39 (22.9)	36 (10.5)
Total	513 (100.0)	170 (100.0)	343 (100.0)
Race, *n* (%)^b^			
Asian	35 (6.8)	9 (5.3)	26 (7.5)
Black/Hispanic	11 (2.1)	6 (3.5)	5 (1.5)
Caucasian	422 (82.3)	146 (85.9)	276 (80.5)
Other	45 (8.8)	9 (5.3)	36 (10.5)
Total	513 (100.0)	170 (100.0)	343 (100.0)
Weight category, kg, *n* (%)			
40 to <50	29 (2.3)	18 (3.8)	11 (1.4)
50 to <64	198 (15.9)	68 (14.5)	130 (16.8)
64 to <75	272 (21.9)	86 (18.4)	186 (24.0)
75 to <85	298 (24.0)	107 (22.9)	191 (24.7)
≥85	444 (35.7)	189 (40.4)	255 (32.9)
Not available	1 (0.1)	0 (0.0)	1 (0.1)
Total	1242 (100.0)	468 (100.0)	774 (100.0)
Metavir fibrosis score, *n* (%)			
F0/F1	150 (12.1)	72 (15.4)	78 (10.1)
F2	134 (10.8)	58 (12.4)	76 (9.8)
F3	89 (7.2)	36 (7.7)	53 (6.8)
F4	62 (5.0)	19 (4.1)	43 (5.6)
Not available	807 (65.0)	283 (60.5)	524 (67.7)
Total	1242 (100.0)	468 (100.0)	774 (100.0)
HCV RNA, log_10_ IU/mL^c^			
Mean (SD)	6.2 (6.5)	6.3 (6.6)	6.1 (6.4)
Median	5.9	5.9	5.8
Range	2.7–7.6	2.7–7.6	2.8–6.5
Length of treatment (weeks), *n* (%)			
0 to <12	47 (3.8)	20 (4.3)	27 (3.5)
12–16	24 (1.9)	7 (1.5)	17 (2.2)
17 to <23	31 (2.5)	9 (1.9)	22 (2.8)
24	979 (78.8)	377 (80.6)	602 (77.8)
>24 to 47	64 (5.2)	26 (5.6)	38 (4.9)
48	15 (1.2)	2 (0.4)	13 (1.7)
Not available	82 (6.6)	27 (5.8)	55 (7.1)
Total	1242 (100)	468 (100.0)	774 (100.0)
Geographic region, *n* (%)			
British Columbia	256 (20.6)	103 (22.0)	153 (19.8)
Atlantic Canada^d^	34 (2.7)	20 (4.3)	14 (1.8)
Ontario	581 (46.8)	229 (48.9)	352 (45.5)
Prairie provinces^e^	188 (15.1)	77 (16.5)	111 (14.3)
Quebec	183 (14.7)	39 (8.3)	144 (18.6)
Total	1242 (100.0)	468 (100.0)	774 (100.0)

*ITT* intention-to-treat; *SD* standard deviation

^a^ *n* = 1242

^b^Data available for RediPEN only; *n* = 513

^c^ *n* = 761

^d^Atlantic Canada includes New Brunswick, Newfoundland and Labrador, and Nova Scotia

^e^Prairie provinces include Alberta, Manitoba, and Saskatchewan

Regarding disease parameters (Table 2), the mean (SD) baseline HCV RNA levels were 6.2 (6.5) log_10_ IU/mL in the ITT population, 6.3 log_10_ IU/mL (6.6) in the G2-infected population and 6.1 log_10_ IU/mL (6.4) in the G3-infected population. Among patients with available Metavir fibrosis stage (ITT: *n* = 432; G2: *n* = 185; G3: *n* = 250), F0/F1 were the predominant stages (34.5 %, 38.9 %, and 31.2 % in the ITT, G2-, and G3-infected populations, respectively), followed by F2 (30.8 %, 31.4 %, and 30.4 %, respectively), F3 (20.4 %, 19.4 %, and 21.2 %, respectively), and F4 (14.3 %, 10.3 %, and 17.2 %, respectively). Treatment duration was predominantly for 24 weeks in the ITT, G2, and G3 populations (78.8 %, 80.6 %, and 77.8 %, respectively).

### Effectiveness

The EVR rate, assessed in RediPEN only (*n* = 513), was 17.3 % (*n* = 89), 18.2 % (*n* = 31), and 16.9 % (*n* = 58) in the ITT, G2-, and G3-infected populations, respectively. EOT response was achieved in 79.1 % of ITT (*n* = 982), 82.5 % of G2- (*n* = 386), and 77.0 % of G3-infected patients (*n* = 596), and SVR in 67.6 % (*n* = 840), 74.4 % (*n* = 348), and 63.6 % (*n* = 492) of patients, respectively (Table 3). Relapse occurred in 16.6 % (*n* = 163) of patients in the ITT population achieving EOT response, and in 12.7 % (*n* = 49) and 19.1 % (*n* = 114) of G2- and G3-infected patients achieving EOT response, respectively (Table 4). SVR and relapse were assessed in the ITT, G2-, and G3-infected populations per gender, race, weight, baseline viral load, geographic location, and fibrosis score. EVR and EOT response were also assessed per genotype per fibrosis score.

**Table 3:** Subgroup analysis for SVR

Variable	Overall ITT (*n* = 1242)			Genotype 2 (*n* = 468)			Genotype 3 (*n* = 774)		
	Yes, *n* (%)	No, *n* (%)	*p*-Value	Yes, *n* (%)	No, *n* (%)	*p*-Value	Yes, *n* (%)	No, *n* (%)	*p*-Value
Overall SVR^a^	840 (67.6)	402 (32.4)	–	348 (74.4)	120 (25.6)	–	492 (63.6)	282 (36.4)	–
Genotype (G)									
2	348 (74.4)	120 (25.6)	<0.001*	–	–	–	–	–	–
3	492 (63.6)	282 (36.4)							
Gender^b^									
Male	203 (63.8)	115 (36.2)	0.082*	73 (72.3)	28 (27.7)	0.119*	130 (59.9)	87 (40.1)	0.342
Female	139 (71.3)	56 (28.7)		57 (82.6)	12 (17.4)		82 (65.1)	44 (34.9)	
Race^b^									
Asian	27 (77.1)	8 (22.9)	0.137*	8 (88.9)	1 (11.1)	0.700	19 (73.1)	7 (26.9)	0.010*
Black/Hispanic	6 (54.5)	5 (45.5)		5 (83.3)	1 (16.7)		1 (20.0)	4 (80.0)	
Caucasian	274 (64.9)	148 (35.1)		111 (76.0)	35 (24.0)		163 (59.1)	113 (40.9)	
Other	35 (77.8)	10 (22.2)		6 (66.7)	3 (33.3)		29 (80.6)	7 (19.4)	
Weight, kg^b^									
40 to <50	22 (75.9)	7 (24.1)	0.869	13 (72.2)	5 (27.8)	0.916	9 (81.8)	2 (18.2)	0.536
50 to <64	134 (67.7)	64 (32.3)		49 (72.1)	19 (27.9)		85 (65.4)	45 (34.6)	
64 to <75	180 (66.2)	92 (33.8)		63 (73.3)	23 (26.7)		117 (62.9)	69 (37.1)	
75 to <85	204 (68.5)	94 (31.5)		78 (72.9)	29 (27.1)		126 (66.0)	65 (34.0)	
≥85	300 (67.6)	144 (32.4)		145 (76.7)	44 (23.3)		155 (60.8)	100 (39.2)	
Not available^c^	0 (0.0)	1 (100.0)		0 (0.0)	0 (0.0)		0 (0.0)	1 (100.0)	
Metavir fibrosis score^d^									
F0/F1	112 (74.7)	38 (25.3)	0.014*	55 (76.4)	17 (23.6)	0.844	57 (73.1)	21 (26.9)	0.003*
F2	83 (61.9)	51 (38.1)		43 (74.1)	15 (25.9)		40 (52.6)	36 (47.4)	
F3	60 (67.4)	29 (32.6)		25 (69.4)	11 (30.6)		35 (66.0)	18 (34.0)	
F4	33 (53.2)	29 (46.8)		15 (78.9)	4 (21.1)		18 (41.9)	25 (58.1)	
Not available^c^	552 (68.4)	255 (31.6)		210 (74.2)	73 (25.8)		342 (65.3)	182 (34.7)	
HCV RNA baseline (log_10_ IU/mL)									
Low: ≤5	75 (72.8)	28 (27.2)	0.178	29 (82.9)	6 (17.1)	0.336	46 (67.6)	22 (32.4)	0.238
High: >5	435 (66.1)	223 (33.9)		194 (75.5)	63 (24.5)		241 (60.1)	160 (39.9)	
Not available^c^	330 (67.6)	151 (32.4)		125 (71.0)	51 (29.0)		205 (67.2)	100 (32.8)	
Geographic location									
British Columbia	183 (71.5)	73 (28.5)	0.645	77 (74.8)	26 (25.2)	0.473	106 (69.3)	47 (30.7)	0.277
Atlantic Canada^e^	23(67.6)	11 (32.4)		15 (75.0)	5 (25.0)		8 (57.1)	6 (42.9)	
Ontario	388 (66.8)	193 (33.2)		174 (76.0)	55 (24.0)		214 (60.8)	138 (39.2)	
Prairie provinces^f^	127 (67.6)	61 (32.4)		51 (66.2)	26 (33.8)		76 (68.5)	35 (31.5)	
Quebec	119 (65.0)	64 (35.0)		31 (79.5)	8 (20.5)		88 (61.1)	56 (38.9)	

*ITT* intention-to-treat; *SVR* sustained virologic response

*Variables included in the multivariate analysis (*p* < 0.150)

^a^% based on the total number of patients with available data for each level of each variable

^b^Data available for RediPEN only; *n* = 513

^c^Not available categories excluded from statistical comparison

**Fig. 1:**
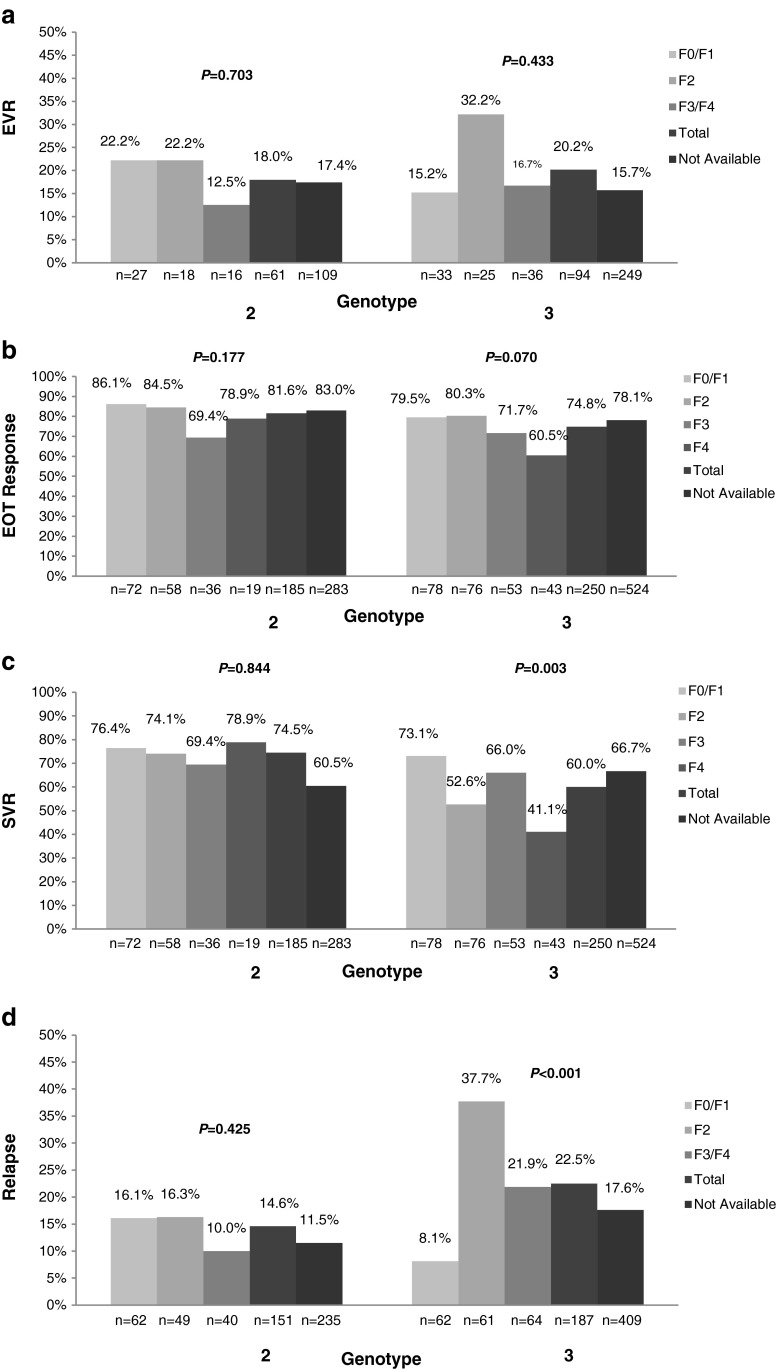
Treatment outcome by genotype per fibrosis score. Proportion of patients by hepatitis C virus (HCV) genotype and baseline Metavir fibrosis score for the following treatment outcomes: early virologic response (EVR) (panel A; collected for RediPEN only: G2 *n* = 170; G3 *n* = 343); end-of-treatment (EOT) response (panel B: G2 *n* = 468, G3 *n* = 774); sustained virologic response (SVR) (panel C: G2 *n* = 468, G3 *n* = 774); relapse (panel D; calculated for patients achieving an EOT response: G2 *n* = 386; G3 *n* = 596). The “Total” category includes all patients with available fibrosis data. Per genotype, the *p*-value for the association between fibrosis stage and treatment outcome is provided. Patients lost to follow-up or with missing EVR, EOT response, and SVR information were considered as non-responders

^d^For the total number of patients with available SVR data per fibrosis level, refer to Fig. 1c

^e^Atlantic Canada includes New Brunswick, Newfoundland and Labrador, and Nova Scotia

^f^Prairie provinces include Alberta, Manitoba, and Saskatchewan

**Table 4:** Subgroup analysis for relapse

Variable	Overall ITT (*n* = 982)			Genotype 2 (*n* = 386)			Genotype 3 (*n* = 596)		
	Yes, *n* (%)	No, *n* (%)	*p*-Value	Yes, *n* (%)	No, *n* (%)	*p*-Value	Yes, *n* (%)	No, *n* (%)	*p*-Value
Overall relapse rate^a^	163 (16.6)	819 (83.4)	–	49 (12.7)	337 (87.3)	–	114 (19.1)	482 (80.9)	–
Genotype (G)									
2	49 (12.7)	337 (87.3)	0.008*	–	–	–	–	–	–
3	114 (19.1)	482 (80.9)							
Gender^b^									
Male	49 (20.4)	191 (79.6)	0.137*	6 (7.8)	71 (92.2)	0.983	43 (26.4)	120 (73.6)	0.118*
Female	22 (14.5)	130 (85.5)		4 (7.7)	48 (92.3)		18 (18.0)	82 (82.0)	
Race^b^									
Asian	4 (13.3)	26 (86.7)	0.827	0 (0.0)	8 (100.0)	0.225	4 (18.2)	18 (81.8)	0.308
Black/Hispanic	1 (16.7)	5 (83.3)		0 (0.0)	4 (100.0)		1 (50.0)	1 (50.0)	
Caucasian	60 (19.0)	256 (81.0)		8 (7.3)	101 (92.7)		52 (25.1)	155 (74.9)	
Other	6 (15.0)	34 (85.0)		2 (25.0)	6 (75.00)		4 (12.5)	28 (87.5)	
Weight, kg^b^									
40 to <50	3 (12.5)	21 (87.5)	0.917	3 (20.0)	12 (80.0)	0.490	0 (0.0)	9 (100.0)	0.623
50 to <64	28 (17.4)	133 (82.6)		10 (17.2)	48 (82.8)		18 (17.5)	85 (82.5)	
64 to <75	39 (18.2)	175 (81.8)		11 (15.3)	61 (84.7)		28 (19.7)	114 (80.3)	
75 to <85	38 (16.0)	199 (84.0)		9 (10.7)	75 (89.3)		29 (19.0)	124 (81.0)	
≥85	55 (15.9)	291 (84.1)		16 (10.2)	141 (89.8)		39 (20.6)	150 (79.4)	
Not available^c^	0 (0.0)	0 (0.0)		0 (0.0)	0 (0.0)		0 (0.0)	1 (0.1)	
Metavir fibrosis score^d,e^									
F0/F1	15 (12.1)	109 (87.9)	0.006*	10 (16.1)	52 (83.9)	0.633	5 (8.1)	57 (91.9)	<0.001*
F2	31 (28.2)	79 (71.8)		8 (16.3)	41 (83.7)		23 (37.7)	38 (62.3)	
F3/F4	18 (17.3)	86 (82.7)		4 (10.0)	36 (90.0)		14 (21.9)	50 (78.1)	
Not available^c^	99 (15.4)	545 (84.6)		27 (11.4)	208 (88.6)		72 (17.6)	337 (82.4)	
HCV RNA baseline (log_10_ IU/mL)									
Low: ≤5	13 (14.9)	74 (85.1)	0.651	2 (6.7)	28 (93.3)	0.334	11 (19.3)	46 (80.7)	0.922
High: >5	85 (16.9)	418 (83.1)		27 (12.8)	184 (87.2)		58 (19.9)	234 (80.1	
Not available^c^	65 (16.6)	327 (83.4)		20 (13.8)	125 (86.2)		45 (18.2)	202 (79.2)	
Geographic location									
British Columbia	34 (15.8)	181 (84.2)	0.945	13 (14.6)	76 (85.4)	0.290	21 (16.7)	105 (83.3)	0.945
Atlantic Canada^f^	5 (17.9)	23 (82.1)		3 (16.7)	15 (83.3)		2 (20.0)	8 (80.0)	
Ontario	71 (16.0)	372 (84.0)		19 (10.3)	165 (89.7)		52 (20.1)	207 (79.9)	
Prairie provinces^g^	29 (17.8)	126 (81.3)		12 (19.0)	51 (81.0)		17 (18.5)	75 (81.5)	
Quebec	24 (17.0)	117 (83.0)		2 (6.3)	30 (93.8)		22 (20.0)	87 (79.8)	

*ITT* intention-to-treat

*Variables included in the multivariate analysis (*p* < 0.150)

^a^% based on the total number of patients with available data for each level for each variable

^b^Data available for RediPEN only; *n* = 513

^c^Not available categories excluded from statistical comparison

^d^Due to the low numbers, F3 and F4 were combined in the univariate and multivariate analyses of relapse

^e^For the total number of patients with available relapse data per fibrosis level, refer to Fig. 1d

^f^Atlantic Canada includes New Brunswick, Newfoundland and Labrador, and Nova Scotia

^g^Prairie provinces include Alberta, Manitoba, and Saskatchewan

#### Subgroup analysis

Figure 1 illustrates the results of the genotype per fibrosis score analysis for EVR, EOT response, SVR, and relapse. Overall, a trend towards improved SVR (Fig. 1c) and relapse (Fig. 1d) was observed in G2-infected patients compared to G3-infected patients. Similarly, patients with low fibrosis showed a better response to treatment, although the impact of fibrosis stage was more evident in patients infected with G3 compared to patients infected with G2 (Fig. 1a–d). Significant associations were observed between fibrosis stage and SVR (F0/F1: 73.1 % vs. F2: 52.6 % vs. F3: 66.0 % vs. F4: 41.9 %; *p* = 0.003) and relapse rate (F0/F1: 8.1 % vs. F2: 37.7 % vs. F3/F4: 21.8 %; *p* < 0.001) in patients with G3 infection but not those with G2 infection.

The results of the detailed SVR subgroup analysis are summarized in Table 3. Overall, significant differences in SVR achievement were observed between genotypes, with G3 being associated with poorer response (74.4 % G2 vs. 63.6 % G3; *p* < 0.001). Fibrosis score (73.1 % F0/F1 vs. 52.6 % F2 vs. 66.0 % F3 vs. 41.9 % F4; *p* = 0.003) and race (73.1 % Asian vs. 59.1 % Caucasian vs. 20.0 % Black/Hispanic; *p* = 0.010) had a significant impact on SVR in the G3-infected population only. No parameters in the G2-infected population were identified as predictors of SVR, although a statistical trend was observed for the association between gender and SVR (male vs. female: 72.3 % vs. 82.6 %; *p* = 0.119). The effect of race on SVR remained unchanged, even upon excluding the “Other” race category.

Table 4 presents the results of the subgroup analysis for relapse. Overall, a significant association was observed between relapse rate and genotype (G2 vs. G3: 12.7 % vs. 19.1 %; *p* = 0.008). The association between fibrosis score and relapse was significant in the G3-infected population (8.1 % F0/F1 vs. 37.7 % F2 vs. 21.9 % F3/F4; *p* < 0.001), while gender was identified as a potential confounder (26.4 % male vs. 18.0 % female; *p* = 0.118). No associations were found for race, weight, baseline HCV RNA levels, and geographic location. Furthermore, no predictors of relapse were identified for the G2-infected population.

#### Multivariate analysis

The results of the multivariate analysis are provided in Table 5. Upon adjusting for potential confounders, genotype was identified as a significant predictor of response in terms of both SVR and relapse: G3-infected patients had approximately 80 % lower odds of achieving SVR [odds ratio (OR) (95 % confidence interval [CI]): 0.20 (0.06–0.64); *p* = 0.007] and nearly 7-fold higher odds of relapsing [OR (95 % CI): 6.84 (1.32–35.34); *p* = 0.022] when compared to G2-infected individuals. Male gender [OR (95 % CI): 14.29 (1.25–125.00); *p* = 0.014] and more advanced fibrosis [F2 vs. F0/F1: OR (95 % CI): 6.79 (1.42–35.52); *p* = 0.017, F3/F4 vs. F0/F1: OR (95 % CI): 2.38 (0.59–13.52); *p* = 0.192] were also identified as independent predictors of relapse in the overall ITT population. Race was not found to have an effect on SVR, irrespective of including or not the “Other” race category.

**Table 5:** Multivariate logistic regression analysis^a^

Population	Outcome	Variable	Level	Odds ratio (95 % CI)	*p*-Value
ITT	SVR	Genotype	Genotype 3 vs. 2	0.20 (0.06–0.64)	0.007
	Relapse^b^	Genotype	Genotype 3 vs. 2	6.84 (1.32–35.34)	0.022
		Gender	Male vs. female	14.29 (1.70–125.00)	0.014
		Fibrosis score	F2 vs. F0/F1	6.79 (1.42–32.52)	0.017
			F3/F4 vs. F0/F1	2.38 (0.59–13.52)	0.192
Genotype 3	SVR^c^	Fibrosis score	F2 vs. F0/F1	0.41 (0.21–0.80)	0.009
			F3 vs. F0/F1	0.72 (0.34–1.53)	0.388
			F4 vs. F0/F1	0.27 (0.12–0.58)	0.001
	Relapse^b^	Gender	Male vs. female	13.16 (1.49–111.11)	0.020
		Fibrosis score	F2 vs. F0/F1	9.72 (1.52–61.99)	0.016
			F3/F4 vs. F0/F1	4.23 (0.71–25.24)	0.113

*CI* confidence interval; *ITT* intention-to-treat; *SVR* sustained virologic response

^a^Significant variables from saturated multivariate models (*p* < 0.05). Multivariate analysis was not conducted for G2 since only gender was identified as a potential predictor in the univariate analysis

^b^Due to the low numbers, F3 and F4 were combined in the relapse analysis

^c^Race was excluded from the model due to numerical instability (low numbers)

With regards to the individual genotypes, given that no significant predictors were identified for G2-infected patients in the univariate analyses (Tables 3 and 4), multivariate analyses were not conducted. However, within the G3-infected population, fibrosis score was identified as a significant predictor of both SVR and relapse: F2 patients had 60 % lower odds of achieving SVR compared to F0/F1 patients [OR (95 % CI): 0.41 (0.21–0.80); *p* = 0.009], F3 patients had 30 % lower odds [OR (95 % CI): 0.72 (0.34–1.53); *p* = 0.388], and F4 patients had over 70 % lower odds [OR (95 % CI): 0.27 (0.12–0.58); *p* = 0.001]. Although, in the univariate analysis, race was found to be significantly associated with SVR, due to instability of the model caused by the low number of patients with available information, it was excluded from the multivariate analysis. Regarding relapse, when compared to F0/F1 individuals, F2 patients had nearly 10-fold higher odds [OR (95 % CI): 9.72 (1.52–61.99); *p* = 0.016] and F3/F4 patients had 4-fold higher odds of relapsing [OR (95 % CI): 4.23 (0.71–25.24); *p* = 0.113]. Relapse was also significantly associated with gender, with male patients having 13-fold higher odds of relapsing than females [OR (95 % CI): 13.16 (1.49–111.11); *p* = 0.020]. No significant independent predictors of EVR or EOT were identified.

### Safety

As observational studies, neither RediPEN nor PoWer were mandated, at the time of study conduct, to collect information on safety. In addition, reasons for discontinuation were recorded only in the RediPEN study (Table 1). Overall, 190 patients discontinued treatment, with adverse events (*n* = 35) being the primary cause in 34 patients [6.6 % of the RediPEN population: 8.8 % (*n* = 15) of G2-infected RediPEN patients and 5.5 % (*n* = 19) of G3-infected RediPEN patients] (Table 1) .

## Discussion

The safety and efficacy of peg-IFN α-2b plus ribavirin for the treatment of chronic HCV infection has been previously evaluated in several controlled clinical trials [7, 11–13, 16, 17, 30–36]. The purpose of this pooled analysis was to separately evaluate, in a large Canadian cohort, the real-world effectiveness of PEGETRON® for the treatment of chronic HCV infection in treatment-naïve individuals with G2 and G3 HCV infection. Observational studies are essential in order to demonstrate the true population-based benefits. In addition, predictors of treatment response were evaluated.

Per genotype, G3 was associated with lower odds of achieving SVR and higher odds of relapse compared to G2. These results are in line with previous reports that have found differences in therapeutic outcomes between G2- and G3-infected populations, where G3 infection has been associated with attenuated treatment responses and higher rates of relapse compared to G2 infection [7, 11–14, 33, 37]. Furthermore, among G3-infected patients, a significant association was observed between advanced liver disease (indicated by more severe fibrosis) and response to treatment. These findings are consistent with previous reports indicating that advanced liver disease is a predictor of higher treatment failure and lower SVR rates [7, 33, 37], especially in G3-infected patients: Powis et al. demonstrated that cirrhotic G3-infected patients experience lower SVR rates (17 % vs. 78 %; *p* = 0.027) [7, 37] than their cirrhotic G2-infected counterparts, and Aghemo et al. reported higher rates of treatment failure in cirrhotic versus non-cirrhotic G3-infected individuals [OR (95 % CI): 10.1 (2.4–41.7)] [37].

Interestingly, significant associations between gender and relapse were observed in both the ITT and G3-infected populations. Although numerous reports have identified female gender as an independent predictor of spontaneous viral clearance [38–42], limited evidence is available for the association between gender and treatment outcomes: while Manns et al. have previously identified an association between gender and SVR [33], numerous publications have failed to identify gender as an independent predictor of response to peg-IFNα + RBV combination therapy [7, 11–13]. However, in individuals with chronic HCV infection, more rapid rates of fibrosis progression have been found in men [43–46], with evidence supporting beneficial effects of estrogen slowing the progression of liver fibrosis in women [47, 48]. In addition, Hayashi et al. found that, although there was no significant gender difference in the overall response to IFN-α monotherapy, being a younger woman (<40 years) was a favorable marker for successful treatment [49], findings substantiated by subsequent data associating menopause with a significant decrease in treatment response and acceleration of liver fibrosis, when compared to women of reproductive age and, thus, higher estrogen levels [45, 47].

In accordance with the observation nature of the RediPEN and PoWer studies, this pooled analysis was limited primarily by the incomplete baseline data regarding race, fibrosis score, gender, and weight, which were only available for the RediPEN study. Viral load data were also incomplete, with 40 % or more of patients missing this information at baseline. Previous studies have identified race as a significant risk factor for poor response to treatment due to a higher prevalence of favorable allelic variations at the *IL28B* promoter in certain ethnic populations [14, 32, 34, 50], and baseline viral load has previously been identified as a predictor of treatment response [7, 11, 13, 14, 30]. Also, a major limitation of the study was the lack of RVR data collected. RVR has been shown to predict treatment response in G2 and G3 patients treated with peg-IFNα + RBV combination therapy, where, in those patients achieving RVR, shorter treatment regimens (12–16 weeks) have been found to be as effective as the standard 24-week treatment [11, 30, 34, 36]. However, as best practice to shorten HCV therapy based on prognostic indicators, including RVR, was not dictated by Canadian HCV treatment guidelines until 2007, the lack of RVR data collected is an accurate reflection of the treatment algorithms advocated at the time of the conduct of both studies [51]. This is corroborated by the less than 2 % of patients overall who received an abbreviated 12–16-week treatment regimen, with the majority of patients receiving the full 24 weeks recommended (78.8 % in the ITT population).

An additional limitation of the current analysis is the lack of steatosis data. Especially important in patients with G3 [8–10], steatosis has been associated with higher rates of relapse in this patient subgroup [52, 53]. As such, we were unable to assess the contribution of steatosis to treatment outcomes, or lack thereof, in G3 patients. Also, assessment of the IL28 genetic polymorphism, a known predictor of treatment response, was not part of the two studies analyzed. However at the time of the conduct of the studies, the genetic contribution to anti-HCV treatment response, specifically IL28 [54, 55], was not characterized, and, thus, genotyping patients was not standard of care [51]. Furthermore, due to the fact that, at the time of study conduct, the collection of adverse events during observational research was not mandated by regulatory agencies, all adverse events documented pertain to treatment discontinuation alone, the extent of missing information does not permit a comprehensive safety analysis.

In addition, we report a strikingly lower proportion of patients experiencing EVR than the approximately 90 % EVR rates reported for G2/G3 patients in the literature [30, 56, 57]. However, due to the ITT approach taken by the current analysis, all patients lost to follow-up, or with missing EVR, EOT response, and SVR information, were considered as non-responders. As such, EVR, which was collected for RediPEN only, was available for 23.2 % of patients (*n* =119). However, when calculated based on the number of patients with available data, EVR occurred in 89.9 % of patients overall, and in 94.7 % of G2 and 87.7 % of G3 patients. These values are comparable to those reported in the literature, where a study by Shiffman et al. found EVR rates of 89 % overall (G2 and G3 combined), and in 94 % of G2 and 85 % of G3 patients, treated with PEG/RBV for 24 weeks, respectively [30]. Although this may be an over-estimation of EVR, the high proportion of patients with missing data may be used to rationalize a less conservative approach in the discussion of this outcome.

Nonetheless, the external validity of our findings is not compromised, as we provide evidence from a large cohort supporting, in a real-world setting, the effectiveness of peg-IFN α-2b plus ribavirin in the treatment of chronic HCV G2 and G3 infections. In addition, our data not only substantiate recent evidence identifying G3 as a poor prognostic marker when compared to G2, but also confirm fibrosis and indicate gender as important predictors of treatment outcome in G3-infected patients.

Up until recently, treatment guidelines were unanimous in their recommendations of dual combination therapy for the treatment of HCV G2–6 infections, and triple combination therapy for G1 infection. However, the 2014 recommendations put forth by the American Association for the Study of Liver Diseases (AASLD), as well as the most recent guidelines from the Canadian Association for the Study of the Liver (CASL) recommend first-line SOF dual combination therapy with ribavirin for the treatment of G2 and G3 [24, 25]. Although SVR rates achieved are promising [21–23], the advantage of SOF over PEG/RBV is restricted by its cost, as the price tag on a 12-week course of therapy is CAD $55,000 and USD $84,000, both of which exclude the price of ribavirin [58]. Furthermore, both the AASLD and CASL guidelines recommended a 12-week course of treatment for G2 infections, and 24 weeks for G3 infections, doubling the projected costs for G3-infected patients [24, 25].

The outcomes of several recent cost-effectiveness analyses have found that SOF regimens at their current costs, specifically with regards to the treatment of treatment-naïve, non-cirrhotic G2 and G3 patients, are less cost-effective than PEG/RBV when considering ICERs in the context of WTP thresholds of up to $100,000 [26–29]. Furthermore, in order to achieve cost-effectiveness in G2 and G3 patients, Najafzadeh et al. report that the cost of SOF-containing regimens would have to cost less than $4500 and $5500 dollars a week, respectively [28]. Based on the results of the current analysis and taking into consideration the proven clinical and cost effectiveness of peg-IFN α plus ribavirin for the treatment of HCV [59], treatment with PEG/RBV remains an effective and viable alternative in subsets of G2 and G3 HCV-infected patients, particularly in treatment-naïve individuals with lower fibrosis scores, and reimbursement limitations.
